# Natural infection of phlebotomines (Diptera: Psychodidae) by *Leishmania* (*Leishmania*) *amazonensis* in an area of ecotourism in Central-Western Brazil

**DOI:** 10.1186/s40409-015-0041-8

**Published:** 2015-10-01

**Authors:** Andreia Fernandes Brilhante, Vânia Lúcia Brandão Nunes, Kleber Augusto Kohatsu, Eunice Aparecida Bianchi Galati, Maria Elizabeth Ghizzi Rocca, Edna Aoba Yassui Ishikawa

**Affiliations:** Department of Epidemiology, School of Public Health, University of São Paulo (USP), Av. Dr. Arnaldo, 715, São Paulo, SP CEP 01246-904 Brazil; Laboratory of Human Parasitology, Anhanguera-Uniderp University, Campo Grande, MS Brazil; Center for Zoonosis Control, Bonito, MS Brazil; Tropical Medicine Nucleus, Pará Federal University, Pará, PA Brazil

**Keywords:** *Bichromomyia flaviscutellata*, *Lutzomyia longipalpis*, Natural infection, *Leishmania (Leishmania) amazonensis*

## Abstract

**Background:**

Bonito municipality, known as an area of ecoturism, in Mato Grosso do Sul state, Brazil, is also a focus of visceral and cutaneous leishmaniases, with cases registered in both human and canine populations. This study sought to investigate natural infection by flagellate forms of *Leishmania* in phlebotomines of the urban area of Bonito.

**Findings:**

Sand flies were collected fortnightly from October 2005 to July 2006 with modified automatic light traps installed in peridomiciles and animal shelters in the center and on the outskirts of the city. The females were dissected and their guts observed under an optical microscope. A total of 1977 specimens were captured, *Lutzomyia longipalpis* (88.4 %) and *Bichromomyia flaviscutelata* (3.0 %) being the most frequent species. *Bi. flaviscutellata* was found infected by flagellates that were identified as *Leishmania (Leishmania) amazonensis* by indirect immunofluorescence reaction, employing monoclonal antibodies and the biotin-avidin system. This is the first report of natural infection by *L. amazonensis* in *Bi. flaviscutellata* in a Brazilian urban area.

**Conclusions:**

As *Bi. flaviscutellata* is only slightly attracted by humans, the transmission of *L. amazonensis* in the study area may have a zoonotic character; however, the sympatric occurrence of this parasite and *Lu. longipalpis* should be taken into consideration by the local health authorities since this sand fly has already been found with *L. amazonensis* DNA in a focus of canine visceral leishmaniasis in Bonito municipality.

## Findings

Leishmaniases are a group of anthropozoonoses caused by protozoan parasites of the genus *Leishmania* (Kinetoplastida: Trypanosomatidae) and transmitted by the bite of several species of phlebotomine sand flies (Diptera: Psychodidae) [1]. The rate of naturally infected sand flies with *Leishmania* in endemic areas and the identification of the parasite in a particular phlebotomine species are important in vectorial and epidemiological studies of leishmaniases [2].

Bonito municipality, an important area of ecotourism due to the commercial exploitation of its great scenic beauty, is also a focus of visceral and cutaneous leishmaniases [3, 4]. Studies of the phlebotomine fauna in its rural and urban environments have revealed several vectors or probable vectors of cutaneous and visceral leishmaniasis agents [3–6]. The aim of this study was to investigate natural infection by *Leishmania* spp. in phlebotomines of the urban area of Bonito.

The study was carried out in the urban area of Bonito (21°07’16”S and 56°28’55”W) located on the Bodoquena Range in the southwestern region of Mato Grosso do Sul state, Brazil. Phlebotomines were collected fortnightly from October 2005 to July 2006 with modified automatic light traps coupled to cages at six sites in nine ecotopes, one of them in the central district and the other five at various points on the outskirts, covering peridomestic areas and animal shelters.

The females were immobilized with ethyl ether and transferred to a slide containing a drop of saline solution. The dissection was performed under a stereoscope to expose their digestive tract and genitalia, which were covered by a cover glass and examined under a microscope to search for flagellates in the gut and identification of the phlebotomine species according to Galati’s classification [7]. The males and undissected females were clarified before the identification. The abbreviations of the names of the phlebotomine species follow Marcondes [8].

When flagellates were observed more saline solution was added to the liquid containing the digestive tract; then liquid together with gut aspirated from the slide were inoculated into the hind paws of two hamsters (*Mesocricetus auratus*). As soon as the lesions developed, fine needle aspirations from these were inoculated into a blood agar base culture medium NNN (Neal, Novy, Nicolle) with the liquid phase consisting of BHI (bovine brain-heart infusion) plus penicillin (1000 IU/mL) and streptomycin sulfate (100 μg/mL). Once the stain was isolated, it was it was sent to the Tropical Medicine Nucleus, Pará Federal University, Belém, and subsequently analyzed by indirect immunofluorescence reaction, employing monoclonal antibodies and the biotin-avidin system [9].

The number of sand fly species collected as well as their sex and ecotope are listed in Table 1. Of these 1977 phlebotomines (1515 males and 462 females), *Lutzomyia longipalpis* (88. 5 %) – the known vector of *Leishmania infantum chagasi* – and *Bichromomyia flaviscutellata* (3.0 %) – the vector of *Leishmania (L.) amazonensis –* were the predominant species. *Lu. longipalpis* presented the highest frequencies in ecotopes close to animal shelters such as hen houses with wooden walls, roofs of palm leaves or plywood and the pigpens fenced with boards. This species was less common in forested areas and hillside savannah. *Bi. flaviscutellata* was captured in a hillside vestigial savannah area about 50 m away from the peridomiciles, which consisted of native plants and fruit trees such as banana, mango, papaya, guava and citrus. *Nyssomyia whitmani,* the recognized vector of *Leishmania* (*Viannia*) *braziliensis,* presented a low frequency (1.6 %) and was collected only on the urban perimeter on forested slopes and in peridomiciles surrounded by savannah.

**Table 1:** Phlebotomines distribution according to species, sex and the ecotopes where the traps were installed in Bonito municipality, Mato Grosso do Sul state, Brazil, from October 2005 to July 2006

Trap		T1		T2		T3		T4		T5		T6		T7		T8		T9		Subtotal		Total	
Species	Sex	M	F	M	F	M	F	M	F	M	F	M	F	M	F	M	F	M	F	M	F	MF	%
*Bi. flaviscutellata*		–	15	–	27	–	–	–	1	–	–	–	–	6	11	–	–	–	–	6	54	60	3.0
*Br. avellari*		8	10	4	3	–	–	–	–	–	–	–	–	–	–	–	–	–	–	12	13	25	1.3
*Br. brumpti*		13	21	3	9	1	–	–	–	–	–	–	–	–	2	–	–	1	1	18	33	51	2.6
*Ev. corumbaensis*		–	6	–	2	–	–	–	–	–	–	–	–	–	–	–	–	–	–	–	8	8	0.4
*Ev. sallesi*		–	11	2	7	–	3	3	4	–	–	–	–	2	2	1	–	–	–	8	27	35	1.8
*Lu. longipalpis*		6	3	6	1	107	28	472	92	69	8	10	2	732	146	46	12	3	5	1451	297	1748	88.4
*Mi. quinquefer*		–	1	–	–	–	–	1	–	–	–	–	–	–	–	–	–	–	–	1	1	2	0.1
*Ny. whitmani*		4	12	2	3	–	1	–	–	–	–	–	–	4	6	–	–	–	–	10	22	32	1.6
*Pa. aragaoi*		4	1	–	1	–	–	–	–	–	–	–	–	–	–	–	–	–	–	4	2	6	0.3
*Pa. campograndensis*		–	1	–	–	–	–	–	–	–	–	–	–	–	–	–	–	–	–	–	1	1	0.05
*Pa. punctigeniculata*		*–*	*–*	*–*	1	*–*	–	–	–	–	–	–	–	–	–	–	1	–	–	–	2	2	0.1
*Pa. bigeniculata*		*–*	1	2	–	–	–	–	–	–	–	–	–	3	–	–	–	–	–	5	1	6	0.3
*Pi. christenseni*		*–*	1	–	–	–	–	–	–	–	–	–	–	–	–	–	–	–	–	–	1	–	0.05
Total		35	83	19	54	108	32	476	97	69	8	10	2	747	167	47	13	4	6	1515	462	1977	100

M: Male; F: Female. T1 and T2 - inside and on the edge of a relatively well preserved fragment of deciduous forest, in Vila Machado district, on the west side of Bonito, between two hotels. T3 - on a smallholding, in Vila América district, close to a pigpen. T4 - in a peridomicile in Vila América district, close to a hen house. T5 and T6 - in a peridomicile, close to a hen house, and in a house in the central district. T7 and T8 - in a peridomicile of a smallholding, near a pigpen and an animal shelter, and in a domicile in Vila Alvorada district, in the north of Bonito, close to the central district. T9 - in vestigial hillside savannah in Vila Donária district, in the south-west of Bonito, close to some hotels and to the central district. *Bi*.: *Bichromomyia*; *Br*.: *Brumptomyia*; *Ev*.: *Evandromyia*; *Lu*.: *Lutzomyia*; *Mi*.: *Micropygomyi*a; *Ny*.: *Nyssomyia*; *Pa*.: *Psathyromyia* and *Pi*.: *Pintomyia*

Of 462 females collected, 430 (93.1 %) were dissected. Suprapylarian natural infection by flagellates was found in one female *Bi. flaviscutellata* (1.9 %) and inoculated into two hamsters. After five weeks, cutaneous lesions were present in both the hind paws of the animals from which the strain was isolated and subsequently identified by immunofluorescence reaction, employing monoclonal antibodies and the biotin-avidin system, as *L. (L.) amazonensis*.

*Leishmania (L.) amazonensis* causes cutaneous and occasionally anergic diffuse cutaneous leishmaniasis in individuals with faulty cell-mediated immunity and is widely distributed in Latin America [1]. However, in the Brazilian state of Bahia, it has previously been associated with human visceral leishmaniasis [10]. Although this parasite is predominantly associated with human and animal infection in rural environments [11, 12], it has been found in dogs in Araçatuba municipality in São Paulo state and in domestic cats in urban environments in the municipalities of Campo Grande and Ribas do Rio Pardo in Mato Grosso do Sul state [13–15].

The slight attractiveness of humans to *Bi. flaviscutellata* [16] may perpetuate the transmission of *L. amazonensis* as a zoonotic affliction in the Bonito region. However, *Lu. longipalpis* has its capacity to transmit *L. amazonensis* to hamsters demonstrated experimentally and has already been associated with *L. amazonensis* in a focus of human cutaneous and canine visceral leishmaniases in the Bonito municipality [2, 17].

In the opinion of these authors, the above scientific information should be taken into consideration by the authorities responsible for public health policies in Bonito in their establishment of prevention measures against leishmaniasis, which would include integrated control of vectors and reservoirs, precocious diagnosis of canine and human cases, treatment of human cases and relevant educational measures.

### Ethics committee approval

The present study was not evaluated by the Animal Ethics Committee, because it was performed prior to the publication of the Brazilian law 11.794 of October 2008 laying down procedures for scientific use of animals.
